# Economic Evaluations of mHealth Interventions for the Management of Type 2 Diabetes: A Scoping Review

**DOI:** 10.1177/19322968231183956

**Published:** 2023-07-03

**Authors:** Ida Tornvall, Danelle Kenny, Befikadu Legesse Wubishet, Anthony Russell, Anish Menon, Tracy Comans

**Affiliations:** 1 Centre for Health Services Research, The University of Queensland, Brisbane, QLD, Australia; 2 Centre for Health Services Research, Faculty of Medicine, The University of Queensland, Brisbane, QLD, Australia; 3 Centre for Economic Impacts of Genomic Medicine, Macquarie Business School, Macquarie University, Sydney, NSW, Australia; 4 Department of Endocrinology, Alfred Health, Melbourne, VIC, Australia; 5 School of Public and Preventive Health, Monash University, Melbourne, VIC, Australia; 6 Department of Endocrinology, Metro South Health, Brisbane, QLD, Australia

**Keywords:** mHealth, digital health, type 2 diabetes, health economics, cost-effectiveness

## Abstract

**Background:**

There is plenty of evidence supporting the clinical benefits of mHealth interventions for type 2 diabetes, but despite often being promoted as cost-effective or cost-saving, there is still limited research to support such claims. The objective of this review was to summarize and critically analyze the current body of economic evaluation (EE) studies for mHealth interventions for type 2 diabetes.

**Methods:**

Using a comprehensive search strategy, five databases were searched for full and partial EE studies for mHealth interventions for type 2 diabetes from January 2007 to March 2022. “mHealth” was defined as any intervention that used a mobile device with cellular technology to collect and/or provide data or information for the management of type 2 diabetes. The CHEERS 2022 checklist was used to appraise the reporting of the full EEs.

**Results:**

Twelve studies were included in the review; nine full and three partial evaluations. Text messages smartphone applications were the most common mHealth features. The majority of interventions also included a Bluetooth-connected medical device, eg, glucose or blood pressure monitors. All studies reported their intervention to be cost-effective or cost-saving, however, most studies’ reporting were of moderate quality with a median CHEERS score of 59%.

**Conclusion:**

The current literature indicates that mHealth interventions for type 2 diabetes can be cost-saving or cost-effective, however, the quality of the reporting can be substantially improved. Heterogeneity makes it difficult to compare study outcomes, and the failure to report on key items leaves insufficient information for decision-makers to consider.

## Introduction

### Increasing Burden of Type 2 Diabetes

Diabetes is a major contributor to morbidity and mortality globally and living with diabetes can have a serious impact on the quality of a person’s life. In 2019, diabetes was ranked as the ninth leading cause of death in the world, and the direct cause of 1.5 million deaths.^1^ The World Health Organization (WHO) states that 95% of all diabetes cases are type 2 diabetes mellitus (T2DM),^1^ and an escalating prevalence is increasing pressure on health systems around the world. In 2015 to 2016, $2.7 billion (2.3%) of the Australian health system’s disease expenditure was attributed to diabetes,^2^ but the total cost of diabetes in Australia is estimated to be around $15 billion per year.^3^ These excess costs are largely attributed to the treatment of complications and comorbidities, such as coronary heart disease and kidney disease.^4^

### mHealth

Mobile health (mHealth) is health care supported by mobile technologies. It is a subset of telehealth, which is health care facilitated by telecommunications. While telehealth generally includes synchronous communication between clinicians and patients, mHealth technologies are asynchronous and often automated. The WHO has recognized mHealth technology as an important resource for health services delivery, emphasizing that mHealth improves coverage and quality of care, and increases access to health information.^5^ Thanks to an almost universal uptake of mobile phones and internet coverage, even in regional areas with limited infrastructure,^6^ mHealth provides an opportunity to extend specialist diabetes care from the traditional urban hospitals to underserved regional areas. This is of particular importance as the risk factors associated with type 2 diabetes are more prevalent in non-urban settings.^2^

### Type 2 Diabetes and mHealth

Because of its chronic and complex nature, diabetes is a condition that requires a high level of monitoring and self-management in between health care appointments.^7^ Some examples of essential self-care behaviors for diabetes include daily monitoring of blood glucose levels, medication adherence, healthy eating, and physical activity.^7^ mHealth interventions that can help support these behaviors include text messages and smartphone applications for blood glucose tracking, insulin education and management, and studies have shown that mHealth interventions can have a positive effect on glycemic control in type 2 diabetes by assisting patients with self-monitoring, medication adherence, and behavior change.^8-10^ A systematic review of telehealth interventions for type 2 diabetes by Eberle and Stichling^11^ found that, out of the 99 studies included, 85% had found an explicit beneficial effect because of a telecommunications intervention. In the sub-analysis of asynchronous interventions (eg, text messages), 96% of studies showed an improvement in glycated hemoglobin (HbA1c). In addition, a 2018 Australian systematic review^12^ of smartphone apps for self-management of chronic disease, such as asthma, chronic obstructive lung disease (COPD), heart disease, etc, found that consistent evidence of benefit was only seen with diabetes apps. This further highlights the opportunities of smartphone technology for the management of type 2 diabetes.

### Economic Evaluation Studies

Economic evaluations (EEs) of health technologies help guide decision-makers in determining how to allocate health care funds. The most commonly used EE frameworks in health and medicine are cost-minimization analysis (CMA), cost-effectiveness analysis (CEA), cost-utility analysis (CUA), and cost-benefit analysis (CBA). A CMA compares two or more interventions with similar effects to identify the cheapest alternative^13,14^ and a CBA converts all outcomes to a monetary value. A CEA measures benefits in natural units or clinical outcomes, such as life-years gained^14,15^ and a CUA measures benefits in healthy years, such as quality-adjusted life-years (QALYs).^16^ Cost-effectiveness analysis (CEAs) and CUAs often present their results as an incremental cost-effectiveness ratio (ICER), which is calculated by dividing the difference in total costs (incremental cost) by the difference in health outcome, such as HbA1c (incremental effect).^17^

mHealth interventions are often promoted as potentially cost-effective solutions to health care delivery, particularly for non-communicable diseases, such as type 2 diabetes.^18^ However, despite multiple studies showing clinical promise, EE studies for mHealth for type 2 diabetes are scarce.^8-10^ There is, to the best of our knowledge, only one previous study that has reviewed the costs and cost-effectiveness of mHealth interventions for type 2 diabetes.^19^ This review used a broad definition of “mHealth,” and many of the included interventions did not have a mobile phone component. In this review, we have used a narrower definition of mHealth, only focusing on interventions that included a mobile phone or tablet.

The aim of this review was to scope the literature on EE studies for mHealth interventions for management of type 2 diabetes, with the objective to summarize and critically analyze the existing evidence.

## Study Design

A scoping review framework was chosen for this review as it was better suited for our objective, which was to summarize and critically analyze the existing literature. Heterogeneity—ie, variability in study outcomes—is a common issue in health EEs, making them unsuitable for meta-analysis.^20^ Moreover, mHealth studies can vary greatly between study and intervention designs, leading to further difficulties for comparing outcomes across studies.

The review was guided by the five-stage framework outlined by Arksey and O’Malley,^21^ with methodology guidance from the Joanna Briggs Institute (JBI) *Manual for Evidence Synthesis*.^22^

### Inclusion and Exclusion Criteria

We considered literature referring to adults living with type 2 diabetes. We defined “mHealth” as any intervention that used a mobile device with cellular technology to collect and/or provide data or information for the management of type 2 diabetes. Interventions only including voicecall or videocall components were excluded. Interventions for the prevention of type 2 diabetes were also excluded.

Both full and partial EEs were included. Full evaluations were defined as those that compare both the cost and consequences of at least two treatments, which include studies that follow an accepted evaluation framework, such as CEA, CUA, or CMA.^23^ Partial evaluations were defined as studies that provide a cost measure for the intervention, but no comparison to an alternative intervention or a relation to the effect.^23^

All socioeconomic and geographical settings were considered for inclusion. We only reviewed studies published in English, and only included peer-reviewed and published papers.

### Search Strategy

We searched for literature published in English from January 2007 to March 2022 on PubMed, Embase, CINAHL, Scopus, and EconLit. We chose 2007 as this was the year when the first-generation iPhone was released, which is the device well known for transforming mobile phone technology.

**Figure 1.**
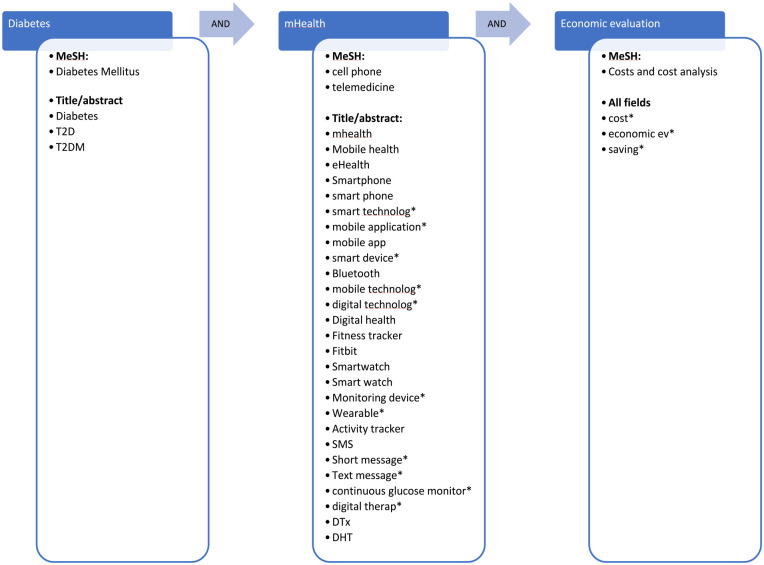
Search string. Synonyms are connected with Boolean operator “OR,” and main categories (blue boxes) are connected with “AND” as indicated by the arrows. Abbreviations: MeSH, medical subject heading; T2D, type 2 diabetes; T2DM, type 2 diabetes mellitus; SMS, short message service, DTx, digital therapeutics; DHT, digital health technology.

The keyword search was divided into three main themes: “diabetes,” “mHealth,” and “economic evaluation.” The search strategy was developed for Medline and modified for each database with verification from a health science librarian. A generalized search string is shown in Figure 1.

### Study Selection and Data Extraction

The results from the database searches were combined in EndNote, where duplicates were removed. The EndNote library was then exported and uploaded into Covidence, which is a browser-based systematic review management software (app.covidence.org, 2022 version), where further duplicates were identified and removed. The first reviewer screened all titles and abstracts against the selection criteria and excluded irrelevant studies. The full texts were then screened independently by the first and the second reviewers. Any disagreements were discussed between the first and the second reviewers and, if consensus could not be reached, a third reviewer was consulted.

A data extraction instrument was created, guided by the one used by Rinaldi et al,^19^ in a review with similar scope. Data collected included details about the study design, type and purpose of the mHealth intervention, and details and outcomes of the EE. The data collection instrument can be found in Appendix A.

Currency conversion rates to 2022 US dollars (USD) were calculated using the CCEMG EPPI-Centre Cost Converter (v 1.4).^24^

### Critical Appraisal

Two reviewers critically appraised the full EE studies using the 2022 Consolidated Health Economic Evaluation Reporting Standards (CHEERS) checklist,^25^ a 28-item checklist that was developed by the International Society for Pharmacoeconomics and Outcomes Research (ISPOR) as a guide for good reporting practice (Appendix B). Studies received one score per item met, and half a score if an item was partially met. We considered a score of more than 75% to be high quality, 50% to 75% moderate quality, and a score of 50% or less was considered poor quality. As the CHEERS checklist was developed for full EEs, we chose to exclude partial evaluations from the reporting assessment.

## Results

**Figure 2.**
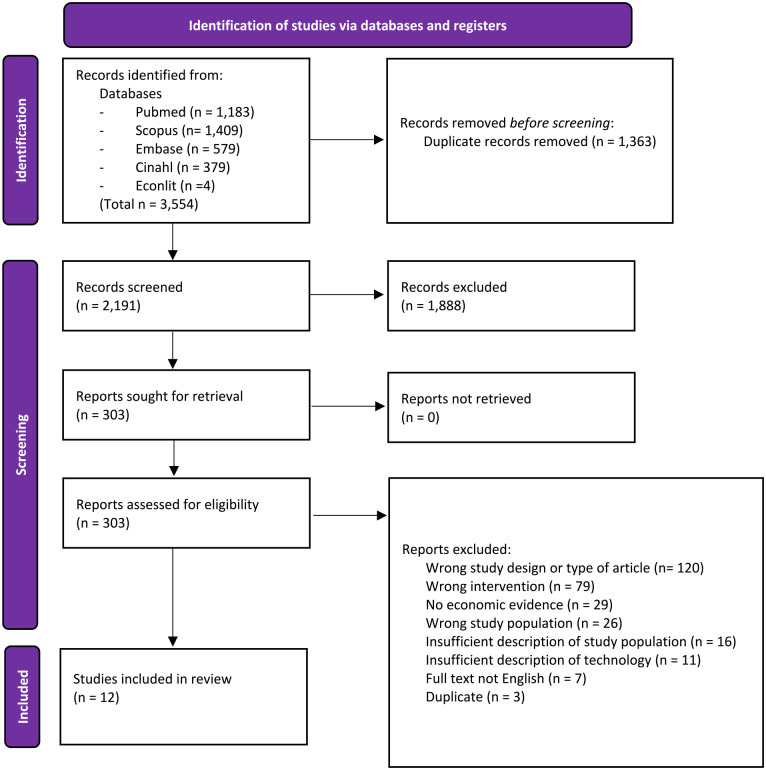
PRISMA 2020 flowchart of the study selection process.

The database search, on March 26, 2022, generated a total of 3554 records. Following the selection process, 12 studies were included in the review. Figure 2 shows the study selection as per the Preferred Reporting Items for Systematic Reviews and Meta-Analyses (PRISMA) 2020 flowchart.^26^

A summary of the characteristics of the included studies is presented in Table 1.

**Table 1.** Summary Characteristics of the Included Studies.

Authors	Publication year	Country (classification)	Type of mHealth component(s)	Comparator
Fritzen et al^27^	2019	Pan-European: France, Germany, Italy, Spain, United Kingdom (HICs)	• Glucose meter with color-range indicator • Smartphone app	Glucose meter with color-range indicator
Warren et al^28^	2018	Australia (HIC)	• Tablet computer • Bluetooth glucometer • Bluetooth blood pressure monitor • Clinician monitoring portal	Usual care
Katalenich et al^29^	2015	United States (HIC)	• SMS or voice messages • Clinician monitoring portal	Usual care Ideal care (ie, best practice/intention-to-treat)
Nordyke et al^30^	2019	United States (HIC)	Digital therapeutics (smartphone application implied)	Usual care
Gilmer et al^31^	2019	Mexico (UMIC)	• Bluetooth-enabled glucose meter • SMS • Clinician monitoring portal	Arm 1: Standard care Arm 2: Multidisciplinary care management (Project Dulce)
Tsuji et al^32^	2020	Japan (HIC)	Continuous glucose monitoring with smartphone app	Retrospective (clinician-facing) continuous glucose monitoring
Shi and Hellmund^33^	2020	United States (HIC)	Flash glucose monitoring with smartphone app	Self-monitoring blood glucose (finger-prick)
Isaacson et al^34^	2020	United States (HIC)	Continuous glucose monitoring with smartphone app	Finger-prick glucometer with mobile app
Islam et al^35^	2020	Bangladesh (LMIC)	SMS	Usual care
Li et al^36^	2021	China (UMIC)	• Smartphone app • Smart wearable medical devices • Patient web platform • Data-sharing cloud platform	Usual care
Jendle et al^37^	2021	Sweden (HIC)	Continuous glucose monitoring with smartphone app	Self-monitoring blood glucose (finger-prick)
Norman et al^38^	2022	United States (HIC)	Continuous glucose monitoring with smartphone app	N/a

Abbreviations: HIC, high-income country; SMS, short message service; UMIC, upper middle-income country; LMIC, lower middle-income country.

### Interventions

The interventions in eight (67%) of the 12 included studies included a smartphone application, three (25%) were short message service (SMS)-based, and the study by Warren et al^28^ included a tablet computer with vital sign monitoring software. The majority of interventions (*n* = 9, 75%) also included a Bluetooth-connected medical device, eg, glucose or blood pressure monitors.

Four (33%) of the 12 studies had an intervention that primarily aimed to promote behavior change or education, and ten (83%) interventions had a glucose monitoring component, including continuous glucose monitoring and flash glucose monitoring. Four (33%) studies included a health care professional (HCP) component, although one was reported to have “minimal” involvement (Katalenich et al^29^).

### Study Design and Outcomes

Of the 12 included studies, nine (75%) were full EEs and three were partial EEs. Using income classifications from the World Bank,^39^ the majority (*n* = 9, 75%) of studies were from high-income countries, but all three studies from middle-income countries (Mexico, China, and Bangladesh) were full EEs. No study from a low-income country was identified for inclusion in this review. While one study was set in a “regional city,” there was no study that focused on a rural population. The majority of the included papers (*n* = 10, 83%) were published in 2019 or later.

Four (33%) of the 12 studies were within-trial analyses alongside randomized control trials (RCT), two (17%) used a mixed randomized control trial and modeling design, and four (33%) were modeling studies using data from the existing literature. The remaining two (17%) studies were costing studies that used existing data but no modeling. The time horizons ranged from six months to lifetime, with slightly more than half (*n* = 7, 58%) of evaluations being for 12 months or less. Four studies reported that their intervention was cost-saving, and the remainder were considered cost-effective when compared with usual care.

All studies measuring effectiveness used change in HbA1c as outcome measure, which is the standard natural unit of measurement in diabetes trials.^40^ The most common comparator was standard/usual care, which refers to the routine care provided in the study setting.

The economic outcome measures and reported outcomes are outlined in Table 2. Of the full EEs, there were five (56%) CUAs using cost per QALY, and three (33%) CEAs using cost per natural unit (such as change in HbA1c). There was also one (11%) cost-minimization study included, which compared the costs of three interventions that were all assumed to have the same effect. No CBA was identified for inclusion in this review. All three partial evaluations were cost comparisons that compared the costs of two interventions but did not relate the costs to any clinical outcomes.

**Table 2.** Economic Characteristics of the Included Studies.

Authors	Publication year	Supplemental Material	Reported economic outcome measure(s)	Currency	Conversion rate (to 2022 USD)	Outcome
Fritzen et al^27^	2019	Supplemental material for this article is available online.	Cost-saving/patient/year Total cost-savings	Euro (EUR)	1.38	**“Substantially” cost-saving:** Over ten years, better glycemic management with the intervention led to cost-savings of: • France: €16.1 million • Germany: €57.8 million • Italy: €30.9 million • Spain: €23.8 million • United Kingdom: €5.8 million considering all insulin-treated T2DM patients in the respective countries
Warren et al^28^	2018	CEA	ICER (incremental cost per percentage-point change in HbA1c) Average daily cost/participant	Australian dollar (AUD)	0.75	**Cost-effective:** Total costs (health care + intervention) in the intervention group were lower compared with usual care (mean AU$3781 vs AU$4662)
Katalenich et al^29^	2015	CMA	Cost/patient/year	US dollar (USD)	1.14	**Cost-effective:** When compared with usual care and numerically but not statistically less costly than ideal care Intervention cost: US$681.82 PPPY Ideal care cost: US$976 to $1049 PPPY Usual care: US$1121.07 PPPY
Nordyke et al^30^	2019	CUA	Savings/patient/month	US dollar (USD)	1.08	**Cost-effective at total three-year program costs of $6468** (using a willingness-to-pay threshold of US$50 000/QALY) Cost-savings PPPM estimated to be US$83 in the first year, US$174 in the second year and US$178 in third year
Gilmer et al^31^	2019	CUA	ICER = cost/QALY Intervention cost/person	US dollar (USD)	1.08	**Highly cost-effective in long term**: Under time horizons of 15 to 20 years, not cost-effective under time horizons of five to ten years ICER = $2220
Tsuji et al^32^	2020	CUA	ICER = cost/QALY	US dollar (USD)	1.06	**Cost-effective:** The increases in ICER and QALY over 20 US$33 039/QALY, and 0.11 QALY per person, respectively
Shi and Hellmund^33^	2020	Partial evaluation: Cost comparison	Cost/patient/year	US dollar (USD)	1.06	**Substantial cost-savings** with flash monitoring from 3 to 10 tests or more compared with SMBG
Isaacson et al^34^	2020	Partial evaluation: Cost comparison	Savings/patient/month	US dollar (USD)	1.06	**Cost-saving:** Savings were $417 PPPM
Islam et al^35^	2014	CUA	Cost/QALY gained ICER (percentage-point change in HbA1c) Cost/patient	International dollar (Intl$)	1.17	**Potentially cost-effective:** • Additional cost-per-patient averaged intl$24 • ICER = Intl$30 • Intl$2406 per QALY gained
Li et al^36^	2021	CEA	Cost/patient/year ICER (cost/patient/year)	Chinese Yuan (CNYY)	0.3	**Cost-effective:** The mHealth intervention was able to save CNYY 22.02 PPPY (ICER = −22.02)
Jendle et al^37^	2021	CUA	Cost/QALY gained	Swedish krona (SEK)	0.12	**Cost-effective over a lifetime:** (SEK144, 412 per QALY gained)
Norman et al^38^	2022	Partial evaluation: Cost comparison	Savings/patient/month	US dollar (USD)	1	**Cost-saving:** Average diabetes-related costs decreased by US$424 PPPM after initiating rtCGM

Abbreviations: EE, economic evaluation; CEA, cost-effectiveness analysis; T2DM, type 2 diabetes mellitus; ICER, incremental cost-effectiveness ratio; CMA, cost-minimization analysis; PPPY, per patient per year; CUA, cost-utility analysis; QALY, quality-adjusted life year; PPPM, per patient per month; SMBG, self-monitoring blood glucose; rtCGM, real-time continuous glucose monitoring.

The reported intervention costs are outlined in Table 3. Only the study by Islam et al^35^ mentioned development costs for the mHealth intervention, however, these were excluded from the economic analysis.

**Table 3.** mHealth Component Cost Inclusions.

Authors	Development costs	Cost of device	Software	Ongoing maintenance	Notes
Fritzen et al^27^	No	Yes	No	No	“Device” in this case is the glucose meter that was included in both intervention + comparator. The specific mHealth component was an app; no costs outlined for this
Warren et al^28^	No	Not clear	Not clear	Yes	
Katalenich et al^29^	No	No	Not clear	Yes	
Nordyke et al^30^	No	No	No	No	No intervention costs included in simulation
Gilmer et al^31^	No	Yes	Not clear	No	
Tsuji et al^32^	No	No	No	No	No intervention costs included in Markov’s model
Shi and Hellmund^33^	N/a^a^	Yes	N/a^a^	Yes^a^	Freestyle Libre
Isaacson et al^34^	N/a^a^	No	N/a^a^	No^a^	Dexcom 6
Islam et al^35^	Excluded from analysis	N/a (SMS only)	Yes (cost per SMS)	Yes	
Li et al^36^	No	Not clear	Not clear	Not clear	
Jendle et al^37^	N/a^a^	Yes	N/a^a^	Yes^a^	Freestyle Libre
Norman et al^38^	N/a	N/a	N/a	N/a	This study only focused on the patients’ medical costs

Abbreviation: SMS, short message service.

a The intervention items in these studies were proprietary continuous glucose monitors and it was assumed that the development, software, and ongoing maintenance costs were absorbed in the cost of the device and the sensor patches.

The primary reported perspective of the EEs was the health system, which was used by five (42%) of the included studies. The study by Jendle et al^37^ used a societal perspective, two studies, both from the United States, used a private or commercial payer perspective, and four (33%) studies did not report or were unclear about what perspective their analysis was from.

### Critical Appraisal

We critically appraised the reporting of the nine full EEs using the CHEERS 2022 reporting checklist. We found that the reporting of the studies was of varying quality, with the mode being moderate quality (*n* = 4, 44%). Two studies (22%) were of high quality (Jendle et al^37^ and Gilmer et al,^31^) and two studies (22%) were considered to be of poor quality. The median score for the CHEERS checklist was 59%, and the highest score was 86%. A score summary can be found in Table 4, and the individual outcomes of the quality assessment are displayed in Table 5.

**Table 4.** CHEERS Checklist 2022 Score Summary for the Included Articles.

CHEERS 2022 item	No. of articles with full score (*n* = 9)	% articles with full score
1. Title	1	11
2. Abstract	4	44
3. Background and objectives	8	89
4. Health economic analysis plan	0	0
5. Study population	4	44
6. Setting and location	4	44
7. Comparators	7	78
8. Perspective	4	44
9. Time horizon	1	11
10. Discount rate	2	22
11. Selection of outcomes	8	89
12. Measurement of outcomes	8	89
13. Valuation of outcomes	5	56
14. Measurement and valuation of resources and costs	8	89
15. Currency, price date, and conversion	5	56
16. Rationale and description of model	4	44
17. Analytics and assumptions	6	67
18. Characterizing heterogeneity	0	0
19. Characterizing distributional effects	2	22
20. Characterizing uncertainty	7	78
21. Approach to engagement with patients and others affected by the study	0	0
22. Study parameters	5	56
23. Summary of main results	8	89
24. Effect of uncertainty	4	44
25. Effect of engagement with patients and others affected by the study	1	11
26. Study findings, limitations, generalizability, and current knowledge	4	44
27. Source of funding	7	78
28. Conflicts of interest	9	100

Abbreviation: CHEERS, Consolidated Health Economic Evaluation Reporting Standards.

**Table 5.** Result of Reporting Quality Assessment Using the CHEERS 2022 Checklist.

CHEERS 2022 Item	Warren et al^28^	Tsuji et al^32^	Nordyke et al^30^	Li et al^36^	Jendle et al^37^	Islam et al^35^	Gilmer et al^31^	Fritzen et al^27^	Katalenich et al^29^
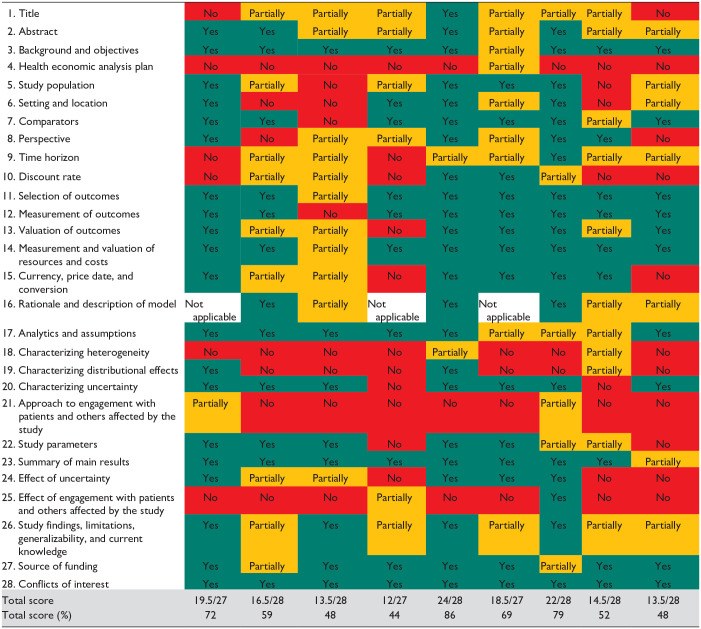									

Abbreviation: CHEERS, Consolidated Health Economic Evaluation Reporting Standards.

Yes = Green, No = Red, Partially = Orange.

Three checklist items, numbers 4, 18, and 21, received no full scores—however, it should be noted that items 4 and 21 are new to the checklist and were not available at the time of publication for the studies in this review. No full scores were given on item 18, Characterizing heterogeneity, as the studies either did not include a subgroup analysis, or failed to describe the methods used for any subgroup analysis.

Only one study (11%) received a full score on item 1: Title, as most papers either did not identify the study as an EE or failed to describe all interventions compared in the study. In addition, only one study (11%) received a full score on item 9: Time horizon. While most studies did outline the time horizon for their analysis, most studies failed to state why it was appropriate.

Furthermore, fewer than half of the studies (44%) received a full score on item 8, Perspective, as several studies either failed to mention the perspective of the economic analysis or failed to relate the perspective to the costs being evaluated. In addition, two of the three partial evaluations included in the review also failed to state or justify the perspective of their analysis.

The only checklist item all studies received a full score on was number 28: Conflict of interest.

## Discussion

This study aimed to scope the literature on current EEs for mHealth interventions for management of type 2 diabetes. We identified 12 studies for inclusion in this review, and all of them indicated that their mHealth intervention was cost-saving or cost-effective. However, when assessing the reporting quality of the included studies, we found the reporting of most papers to be of low or moderate quality.

We found that the included studies differed notably in the type and purpose of their interventions, as well as in study design and outcome measures. Heterogeneity like this is recognized as a common issue in EE studies, making it difficult to compare study outcomes.^20^ In addition, our reporting assessment found that many studies did not report adequately on key items for EEs, such as the perspective, time horizon, or uncertainty of the analysis, which makes it difficult to interpret and generalize the results. This is similar to what Rinaldi et al^19^ concluded in a 2020 systematic review with similar scope, and to the findings of a 2017 systematic review by Iribarren et al^18^ on non-disease specific mHealth interventions.

We also found the reporting of the intervention costs in the reviewed studies to be limited. As shown in Table 3, for some studies, it was unclear what the intervention costs comprised, while other studies chose to exclude the intervention costs from their analysis. The marginal cost for digital health interventions is often assumed to be zero,^41^ however, this is not always accurate since mHealth interventions can include many different components associated with high upfront and ongoing costs, such as hardware, software licenses, and IT support.^42^ Providing detailed information about these costs can help identify drivers of intervention costs and help inform mHealth implementation programs as well as future innovation projects. In addition, the poor generalizability of EE studies in this field due to variations in the intervention designs could also be addressed by more transparent reporting of the intervention costs, as this may provide information relevant to implementation for intervention developers and decision-makers rather than the actual outcomes of a study, which may be setting-specific.

In our screening, we also found that many papers failed to provide sufficient information about the technologies used in the intervention. For example, Nordyke et al,^30^ only describe their assessed intervention as “digital therapeutics,” vaguely implying that they were evaluating a smartphone application. Many studies were therefore excluded at the full-text screening stage with the reason “insufficient description of technology.” It is essential that authors provide adequate description about the evaluated technology to facilitate informed decision-making.

Moreover, while it is positive that all the studies included in this review reported their intervention to be cost-saving or cost-effective, this could also indicate publication bias, as researchers may refrain from publishing negative results. As Iribarren et al^18^ noted, the positive results should be considered with caution, and more research is needed to identify what mHealth technologies have the greatest positive outcomes. Recognizing the limitation of space in research papers, additional information, such as details about the intervention components or cost data, can be supplied as supplementary files, with clear in-text references to these.

Most of the papers included in this review were published in 2019 or later, which indicates that EE studies for mHealth is an emerging field. Half of the included studies were conducted alongside clinical trials, which positively reflects the growing interest in presenting economic information together with the clinical outcomes for new health care technologies.^43^ However, the purpose of EEs is to inform decision-making for resource-allocation,^44^ and when studies fail to report on key items, such as perspective, time horizon, or uncertainty, it could be argued that the economic outcomes are not accurately captured or conveyed. Rather than conducting an economic analysis alongside clinical trials as a box-ticking exercise to satisfy stakeholders, researchers should focus on conducting robust and high-quality economic analyses that both capture and report meaningful information about economic viability. Using a reporting checklist, like the CHEERS 2022 checklist used in this review, can help researchers from medical backgrounds better understand the economic implications of their products and ensure adequate reporting of economic findings.

Despite mHealth having been recognized as a tool to extend health care to underserviced regional and remote areas, and consistent evidence of the clinical benefit of that mHealth interventions for type 2 diabetes, no study included in this review focused on a non-urban population. To facilitate sustainable rollouts of mHealth-facilitated models of care in settings outside urban areas, more economic information is needed. Furthermore, while 80% of people with type 2 diabetes live in low- and middle-income countries (LMICs),^45^ only 25% of the papers included in this study were from a middle-income setting, and there were no studies from low-income countries. Current literature suggests that mHealth programs in LMICs lead to improved clinical outcomes and health behaviors in multiple non-communicable diseases, including diabetes.^46^ While all studies we reviewed from middle-income settings were full EEs, most EE studies for mHealth to date are conducted in high-income countries, and more research is needed to assess the economic viability of mHealth programs in different income settings.

Most full EEs included in this review were CUA and CEA, which are useful frameworks frequently used to evaluate economic impacts of interventions in clinical trials.^43^ A CUA is considered the “gold standard” of health EEs as it uses cost per QALY,^16^ and a CEA is a flexible tool that can measure costs against any clinical outcome. However, due to the perspectives often used in CEAs and CUAs, these evaluations may fail to capture many other benefits of mHealth, such as reduced patient time and travel costs, or increased access to health information. In addition, the use of CEA or CUA limits the outcome to a single unit of measurement, which makes it difficult to measure the combined impact on patients, carers, and health workers.^47^ It has been suggested that a CBA may be a more appropriate evaluation framework for digital health services, as a CBA monetizes all benefits associated with an intervention and can give a more holistic view of an intervention’s benefits.^48^ The WHO recognized the lack of CBAs for mHealth as a gap in the literature in 2011,^49^ and we did not identify any CBA for inclusion in this review. We acknowledge that CBAs are resource-intensive and come with major methodological challenges,^50^ however, the lack of literature in the area still provides an opportunity for more research, particularly in regional and remote areas where the patient-focused benefits may be more evident due to a current lack of service provision.

### Limitations

We acknowledge that this review has some limitations. First, we only included literature published in English. Second, the year 2007 was chosen to capture interventions developed after smartphone technology became available. However, since text messages are an older technology, it is possible we may have missed some studies published prior to our chosen time frame. Third, while we tried to make the process as objective as possible, critical appraisals are generally associated with a certain degree of subjectiveness, and there is a possibility that other researchers may have scored the papers in this review differently on the CHEERS 2022 checklist. And last, “mHealth” is still a fairly ambiguous term, and is often used interchangeably with telehealth, eHealth, and digital health. Other reviews with similar scope to ours may choose to define “mHealth” differently and may thus include different technologies. For example, Rinaldi et. al. defined mHealth interventions as “as interventions that included use of the internet, mobile devices or computers,” while we chose to only include interventions that used a mobile device. Partly as a result of the different definitions, we only included four of the 23 studies from the review by Rinaldi et al. Another reason for the small overlap is that Rinaldi et al also included papers that focused on the prevention for type 2 diabetes, while we only included papers for the management of type 2 diabetes.

As a strategy to overcome the ambiguity in the terminology, we suggest a new medical subject heading (MeSH) is created for “mHealth” in the National Library of Medicine (NLM) library—it is at the time of writing sitting under the “Telemedicine” heading. As the field of digital health is expanding, we would anticipate the digital health MeSHs in the NLM library to evolve and become more defined, making future database searches simpler and more accurate.

## Conclusion

The current literature indicates that mHealth interventions for type 2 diabetes can be cost-saving or cost-effective, however, the quality of the reporting of existing studies can be substantially improved. Heterogeneity in this field, due to interventions often being setting or cohort-specific, makes it difficult to compare interventions and study outcomes, and the failure to report on key information—such as study perspective, time horizon, and description of the intervention—leaves insufficient information for decision-makers to consider. More detailed reporting of intervention components and cost data, including development costs, may improve the generalizability of studies to better inform future projects. More research is also needed to investigate the potential economic benefits of utilizing mHealth for type 2 diabetes management in regional and rural areas, as well as in LMICs.

## Supplemental Material

sj-docx-1-dst-10.1177_19322968231183956 – Supplemental material for Economic Evaluations of mHealth Interventions for the Management of Type 2 Diabetes: A Scoping Review Supplemental material, sj-docx-1-dst-10.1177_19322968231183956 for Economic Evaluations of mHealth Interventions for the Management of Type 2 Diabetes: A Scoping Review by Ida Tornvall, Danelle Kenny, Grad Cert, Befikadu Legesse Wubishet, Anthony Russell, Anish Menon and Tracy Comans in Journal of Diabetes Science and Technology
