# Supplementary material for: Economic Evaluations of mHealth Interventions for the Management of Type 2 Diabetes: A Scoping Review
Source: J Diabetes Sci Technol. 2023 Jul 3;19(1):179–90. doi: 10.1177/19322968231183956 (PMC11688683; doi:10.1177/19322968231183956)
Supplement: sj-docx-1-dst-10.1177_19322968231183956 – Supplemental material for Economic Evaluations of mHealth Interventions for the Management of Type 2 Diabetes: A Scoping Review [file sj-docx-1-dst-10.1177_19322968231183956.docx]

# Appendix A – Data collection instrument

| **Main Category** | **Description** |
| --- | --- |
| Title |  |
| Author(s) |  |
| Publication year |  |
| Country of origin |  |
| Country classification | High/low-middle income |
| Study design | Type of study  Study population  Setting Study objective(s)  Outcome(s)  Comparator |
| Economic evaluation | Type  Perspective  Time Horizon  Outcomes |
| Intervention | Type  mHealth components  Health professional component  Purpose |
| Other key findings relevant to review question |  |

# Appendix B: CHEERS 2022 checklist


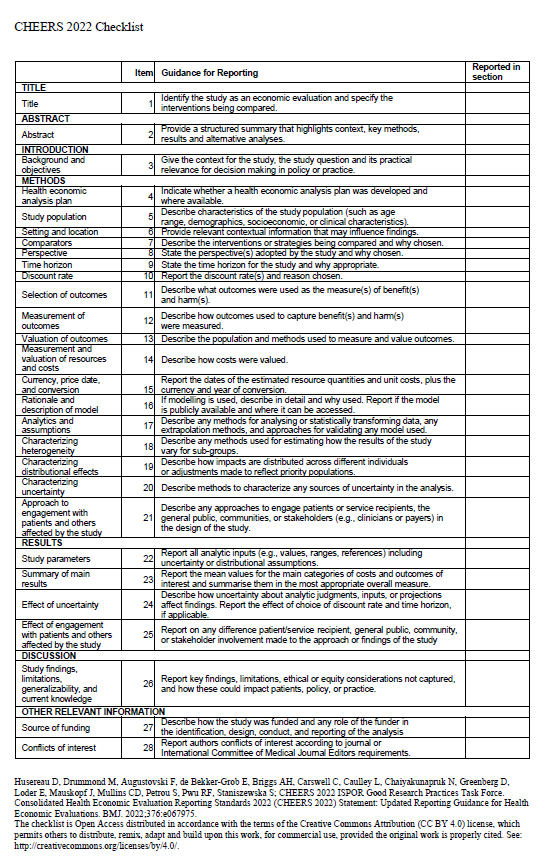
[Image for placeholder, PDF available here: <https://www.equator-network.org/wp-content/uploads/2013/04/CHEERS-2022-checklist-1.pdf> ]
